# Analysis and Description of HOLTIN Service Provision for AECG monitoring in Complex Indoor Environments

**DOI:** 10.3390/s130404947

**Published:** 2013-04-12

**Authors:** Santiago Led, Leire Azpilicueta, Erik Aguirre, Miguel Martínez de Espronceda, Luis Serrano, Francisco Falcone

**Affiliations:** Electrical and Electronic Engineering Department, Edificio Los Tejos, 1 Planta, UPNA, Pamplona, 31006 Navarra, Spain; E-Mails: santiago.led@unavarra.es (S.L.); leyre.azpilicueta@unavarra.es (L.A.); aguirrerik@gmail.com (E.A.); miguel.martinezdeespronceda@unavarra.es (M.M.E.); lserrano@unavarra.es (L.S.)

**Keywords:** ambulatory electrocardiogram, U-Health, HOLTIN, 3D ray launching

## Abstract

In this work, a novel ambulatory ECG monitoring device developed in-house called HOLTIN is analyzed when operating in complex indoor scenarios. The HOLTIN system is described, from the technological platform level to its functional model. In addition, by using in-house 3D ray launching simulation code, the wireless channel behavior, which enables ubiquitous operation, is performed. The effect of human body presence is taken into account by a novel simplified model embedded within the 3D Ray Launching code. Simulation as well as measurement results are presented, showing good agreement. These results may aid in the adequate deployment of this novel device to automate conventional medical processes, increasing the coverage radius and optimizing energy consumption.

## 1. Introduction

Ambulatory Electrocardiogram (AECG) monitoring services are among the most relevant Ubiquitous Health (U-Health) applications due to the high prevalence of cardiovascular disease [1,2]. There are a large number of cardiac pathologies, but healthcare specialists show great interest in diagnosing some of them. Of particular interest are pathologies whose symptoms are palpitations, dizziness and sporadic syncopes, *i.e.*, cardiac conditions that require long-term monitoring systems in order to be diagnosed. The detection of arrhythmic cardiac events (ventricular tachycardia, atrial fibrillation, bradycardia, *etc.*) allows the cardiologist to provide the patient with the most appropiate treatment, usually based on drug administration or pacemaker/ICD implantation. Furthermore, cardiac event detection may be really useful in order to diagnose relevant chronic diseases, such as heart failure. Thus, AECG services that allow continuous and long-term patient monitoring are required to improve diagnosis and treatment of cardivascular diseases. In this sense, cardiologists are very interested in new U-Health services aimed at monitoring of patients that suffer from paroxysmal arrhythmias and sporadic syncopes. Besides from the clinical utility, these new healthcare services should be capable of improving the patient's quality of life [3].

Nowadays, AECG monitoring services used by cardiologists are based on conventional Holter devices and implantable loop recorder systems [4–6]. These systems fulfill the patient's cardiac activity monitoring and allow detecting several types of arrhythmias. However, these systems also present several limitations such as the duration of the monitoring session due to a limited storage capacity and reduced ergonomics. These features may be improved using U-Health approaches. Thus, the new so-called HOLTIN (for INtelligent HOLTer) service has been designed in-house at the Public University of Navarre [7,8]. This service is focused on monitoring of patients that are at low risk, and whose sympthoms are sporadic arrhythmias (ventricular tachycardia, bradycardia), asystolic pauses, and syncopes. Moreover, essential aspects related to development and delivery of U-Health services have been considered in HOLTIN system such as: service goals definition, healthcare professional requirements, technology selection, clinical evaluation, satisfaction of patients, *etc.* A comparative analysis between the HOLTIN service and conventional AECG monitoring systems is shown in Table 1.

The remainder of this paper is organized as follows: Section 2 describes the implementation of the HOLTIN service, with special emphasis on the wearable ECG device used by the patient and its functional model. Section 3 presents the behaviour of the device modeled in a complex radioelectric environment by means of in-house 3D ray launching code coupled to a simplified human body model. Section 4 presents the measurement results obtained in order to validate the performance of the ECG device, as well as to perform a radioplanning analysis of the optimal location within the operational environment of the HOLTIN system. Finally, Section 5 lists the conclusions of the work.

## 2. Description of the HOLTIN Service's Architecture

**Figure 1.**
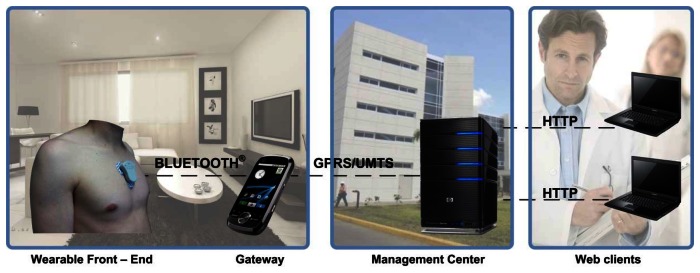
Overview of the HOLTIN platform.

**Figure 2.**
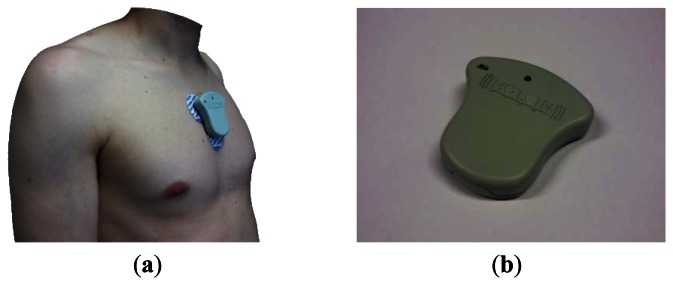
Image of the HOLTIN wearable ECG recorder (**a**) placed on a patient chest (**b**) detail of the device.

The HOLTIN platform consists of a wearable ECG device, a smartphone (data Gateway), a server (data management center) and a set of connecting monitoring clients. The platform shows highly innovative features such as multisystem wireless connectivity, wearable technology and health data management. Besides this technological platform, the service includes a functional model with a detailed description of system operation according to healthcare professional requirements. The elements of the HOLTIN platform are the following (see Figure 1):

- *Wearable ECG recorder*. This device is placed on patient's chest through several disposable wet electrodes avoiding the uncomfortable connection leads used in convetional AECG systems (Figure 2). It performs the ECG waveform (lead II) acquisition, detection of outstanding arrhythmias and its transmission to a Smartphone device via Bluetooth^®^ v2.1 + EDR wireless technology. The ECG recorder has been designed in a small form factor, very low power consumption and high ergonomics in order to improve the patient comfort level.
- *Smartphone*. This gateway device is implemented in a commercial Smartphone with customized service software based on Android 4.0 OS. It receives the acquired ECG data and transmits it to the data management center. This transmission is perfomed via 3G mobile telecomunications technology and a propietary application-level protocol based on commands. The Smartphone also performs tasks related to functional operation of HOLTIN service: mainly ECG recorder association, malfunction warnings, and operation messages.
- *Management center*. This system receives the patients' ECG data and stores it in a database together with demographic information. In this way, healthcare professionals diagnose the patients using online personalized tools.

It is worthwhile to pay attention to several features of the system. On one hand, the use of a wearable acquisition device provides the patient with high comfort and mobility levels. On other hand, the use of a short-range wireless technology for communication between ECG recorder and Smartphone device increases the overall functionality in terms of mobility and battery lifetime. In this sense, Bluetooth^®^ technology provides the whole technical features (frequency hopping spread spectrum, low power consumption, authentication and data encryption, flow control, *etc.*) for being used in this type of system. Although Bluetooth^®^ technology provides a recent release (Bluetooth^®^ v4.0) for very low power applications, the HOLTIN platform uses the Bluetooth^®^ v2.1 + EDR version. It provides a sufficient data rate (up to 3 Mbps) for sending cardiac information with reduced average power consumption and short transmission times; this feature is really important in event recorder devices.

From a functional point of view, the HOLTIN service consists of an extremely elaborated functional model that includes the whole requirements of healthcare staff and takes into account the technological solutions that make possible to fulfill them. ECG recorder performs several operational tasks:

- During the start-up process, the device performs a real time ECG monitoring of the patient in order to allow the healthcare specialist configuring and verify its correct operation. Once the ECG recorder has been initialized, the continuous cardiac event detection process is started.
- The ECG recorder is able to detect and store the patient's outstanding cardiac information in two different operation modes: automatic detection and patient notification. In automatic operation, the device performs a continuous ECG signal processing and detects automatically specific types of cardiac arrhythmic events based on the patient's heart rhythm and several diagnostic settings established by the cardiologist. The device is able to acquire the outstanding data associated to following cardiac events: ventricular tachycardia, bradycardia, and asystolic pauses. In patient notification mode, the patient can trigger a manual event recording process using the Smartphone when he/she feels some arrhythmia symptom (syncope, dizziness). These notifications cause the establishment of Bluetooth^®^ communication between the ECG recorder and the Smartphone for exchanging specific application data.
- The device stores temporarily all detected/notified cardiac events. When storage capacity reaches a specific configurable level, the ECG recorder establishes wireless communication with the Smartphone device in order to transmit all the ECG information. In this way, a permanent Bluetooth^®^ communication with high power requirements is avoided and no relevant patient information is lost.

Although the ECG recorder provides high storage capacity, a reliable wireless communication link is required due to the fact some patients suffer frequent cardiac event episodes. Moreover, this reliability should guarantee the correct operation of the HOLTIN service, independently of the environment where the device is used by the patient. Indoor monitoring is surely one the most regular environments. Thus, it compulsory to model the wireless behavior of the ECG recorder in indoor complex scenarios, where unexpected degradation of the wireless links, especially in the HOLTIN-Smartphone short range communication can occur mainly due to energy absorption and strong multipath components.

## 3. Channel Modeling

Once the HOLTIN ECG recorder has been described, it will be tested to foresee potential limitations derived from the wireless link. For an efficient setup of an indoor wireless sensors network the knowledge of path loss and field coverage in the wireless channel is essential. The behavior of the radio channel in indoor scenarios [9,10] is not a trivial issue and heavily depends of the complexity of the environment. The occurrence of shading effects fundamentally due multipath components but also of phenomenon like reflection, refraction, diffraction and diffuse scattering among others, makes the study of the associated radio channel a complex task. The most straightforward way is to estimate the path loss by means of empirical models based on analytical expressions derived from non-linear regression of the scenario under analysis (*i.e.*, COST 231, Walfish-Bertoni, *etc.*). These models give rapid results, but don't take into account site-specific features related with topology and morphology, require on site calibration and therefore are prone to higher mean error levels as well as higher standard deviation. As an alternative, numerical techniques have been proposed that can fully or partially capture the site-specific features. These methods include ray launching and ray tracing algorithms (based on geometrical approximations) or full wave simulation techniques, such as finite-difference-time-domain (FDTD) [11,12] and pure-full-wave time and frequency domain approaches [13]. These methods are precise, but are time consuming due to inherent computational complexity. As a mid-point, methods based on geometrical optics, for radio planning calculations with strong diffractive elements, offer a reasonable trade-off between precision and required calculation time [14–16].

**Figure 3.**
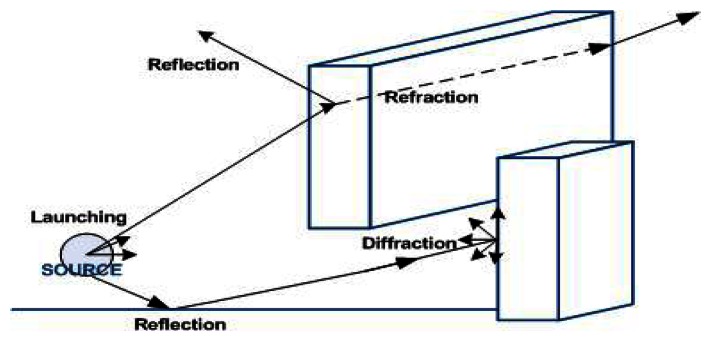
Principle of operation of the 3D ray launching method implemented in-house to perform indoor coverage analysis.

In this work, the estimation of wireless coverage of an indoor scenario has been obtained by means of a 3D ray-launching method for simulating radio wave propagation and penetration. The aim of this analysis is the assessment of the wireless channel between the HOLTIN ECG device and the gateway in terms of capacity and coverage. The algorithm has been implemented in-house at UPNA, based on the Matlab™ programming environment. It is based on Geometrical Optics (GO) and Geometrical Theory of Diffraction (GTD). To complement the GO theory, the diffracted rays are introduced with the GTD and its uniform extension, the Uniform GTD (UTD). The purpose of these rays is to remove field discontinuities and to introduce proper field corrections, especially in the zero-field regions predicted by GO. The principle of the ray launching method is to consider a bundle of transmitted rays that may or may not reach the receiver. The number of rays considered and the distance from the transmitter to the receiver location determines the available spatial resolution and, hence, the accuracy of the model. A finite sample of the possible directions of the propagation from the transmitter is chosen and a ray is launched for each such direction. If a ray hits and object, then a reflecting ray and a refracting ray are generated. If a ray hits a wedge, then a family of diffracting rays is generated, as represented in Figure 3. Rays are launched from the transmitter at an elevation angle θ and with an azimuth angle Φ, as defined in the usual coordinate system. Antenna patterns are incorporated to include the effects of antenna beamwidth in both azimuth and elevation. The material properties for all the elements within the scenario are also taking into account, given the dielectric constant and permittivity at the frequency range of operation of the system under analysis.

A plane electromagnetic wave falling to the planar interface between two regular semi-infinite media 1 and 2 gives rise to two plane waves: reflected and transmitted (or refracted). According to the Snell's law [17], the reflection coefficient *R*^⊥^ and transmission coefficient *T*^⊥^ are calculated by:

(1) $$T^{⊥}=\frac{E_{t}^{⊥}}{E_{i}^{⊥}}=\frac{2\eta_{2}\text{cos}\;\;(\Psi_{i})}{\eta_{2}\text{cos}\;(\Psi_{i})+\eta_{1}\text{cos}\;(\Psi_{t})}$$

(2) $$R^{⊥}=\frac{E_{r}^{⊥}}{E_{i}^{⊥}}=\frac{\eta_{2}\text{cos}\;(\Psi_{i})-\eta_{1}\text{cos}\;(\Psi_{t})}{\eta_{2}\text{cos}\;(\Psi_{i})+\eta_{1}\text{cos}\;(\Psi_{t})}$$

where $\eta_{1}=120\pi /\sqrt{{ɛ}_{r1}}$ , $\eta_{2}=120\pi /\sqrt{{ɛ}_{r2}}$ and Ψ*_i_*, Ψ*_r_* and Ψ*_t_* are the incident, reflected and transmitted angles respectively.

**Figure 4.**
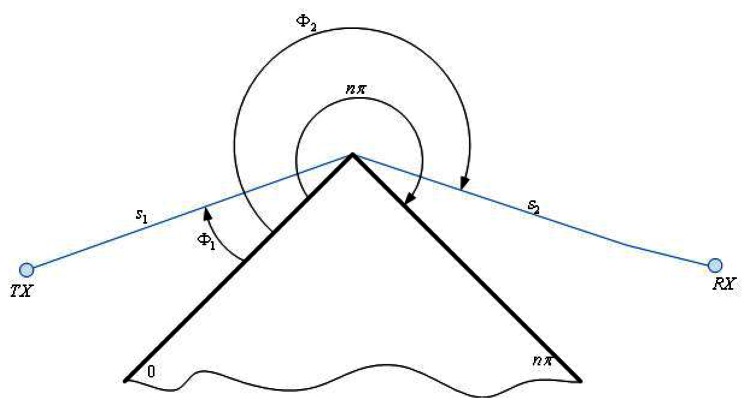
Geometry for wedge diffraction coefficients.

Once the parameters of transmission *T* and reflection *R* are calculated, and the angle of incidence Ψ*_i_* and Ψ*_t_*, the new angles (*θ_r_*, *ϕ_r_*) of the reflected wave and (*θ_t_*, *ϕ_t_*) of the transmitted wave can be calculated. The finite conductivity two-dimensional diffraction coefficients are given by [18,19] as:

(3) $$D^{∥⊥}=\frac{-e^{\left(-j\pi /4\right)}}{2n\sqrt{2\pi k}}\left\{\begin{array}{l} \text{cot}\;g\left(\frac{\pi +(\Phi_{2}-\;\Phi_{1})\;}{2n}\right)F(kLa^{+}(\Phi_{2}-\Phi_{1}))\\ +\text{cot}\;g\;(\frac{\pi -(\Phi_{2}-\Phi_{1})}{2n})F(kLa^{-}(\Phi_{2}-\Phi_{1}))\\ +R_{0}^{∥⊥}\text{cot}\;g\;\left(\frac{\pi -(\Phi_{2}+\;\Phi_{1})}{2n}\right)F(kLa^{-}(\Phi_{2}+\Phi_{1}))\\ +R_{0}^{∥⊥}\text{cot}\;g\left(\frac{\pi +\;(\;\Phi_{2}+\Phi_{1})}{2n}\right)F(kLa^{+}(\Phi_{2}+\Phi_{1})) \end{array}\right\}$$

where *nπ* is the wedge angle, *F*, *L* and *a* ± are defined in [18], *R*_0,*n*_ are the reflection coefficients for the appropriate polarization for the 0 face or n face, respectively. The Φ_2_ and Φ_1_angles in Equation (3) are depicted in Figure 4.

## 4. Results and Discussion

### 4.1. 3D Ray Launching Simulation Results

Simulations and measurements have been performed in a room of the Jerónimo de Ayanz Communications Research Center of the Public University of Navarre.

**Figure 5.**
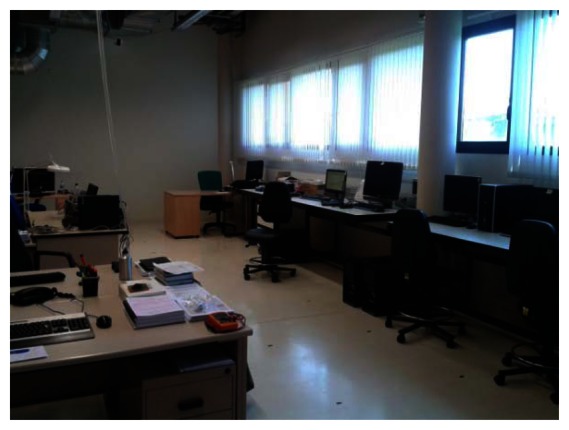
Image of R&D Communications Center laboratory N° 2, in which simulation and measurement results of operation of the HOLTIN system have been performed

The considered scenario, depicted in Figure 5, could be considered as a typical indoor room of a patient's house. It is a complex environment composed of different types of walls (concrete, plywood, *etc.*) and a variety of different furniture (metallic cupboards, tables, chairs, computers, *etc.*) heavily affected by signal degradation due to multipath components. Simulations and measurements have been performed for the HOLTIN transmitter device with a person carrying the transmitter in his chest and afterwards, the device by itself without human body effect.

**Figure 6.**
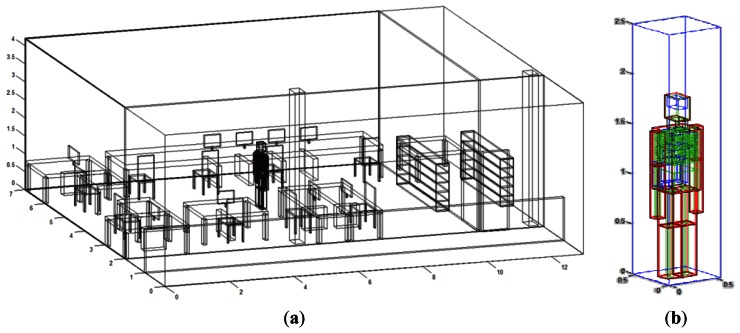
(**a**) R&D Communication's Center laboratory N°2, proposed for deterministic radio channel simulation; (**b**) Detail of the simplified human body model, with the different organs that are embedded within it.

Within the considered indoor scenario, several radiofrequency sources can be placed, in which wireless power is converted into a finite number of rays launched within a solid angle. Parameters such as frequency of operation, radiation diagram of the antennas, number of reflections, separation angle between rays and cuboid dimensions can be fixed. A schematic view of the indoor scenario is shown in Figure 6(a).

In order to fully account for the effect of the presence of patients in the device operation, a simplified human body model has been specifically developed for this 3D ray launching code [20]. This model implements the basic organs considering their frequency dispersive material characteristics, following a Cole-Cole model, in order to analyze their influence on the environment. Figure 6(b) shows a detail of the simplified human body model, which has been performed with the greatest detail as possible, taking into account part such as bones, internal organs, muscles, blood and skin, all with their respective values of dielectric constant and conductivity parameterized to the given frequency range. The human body model has been parameterized in such a way that body proportions (*i.e.*, relative dimensions between head, limbs and torso) are maintained for any given height of the person that is needed to be modeled. The combination of a simplified human body model with an efficient simulation technique enables to assess the impact of wireless systems within the complete scenario under analysis.

Two simulations have been performed for the considered scenario to assess the influence of the presence of a person in the environment, as well as to verify the performance of the HOLTIN transmitter device. The transmitter antenna of the HOLTIN device has an omnidirectional radiation pattern with 1.89 dBi gain. The first simulation was with a simplified human body model located at the center of the room, with the HOLTIN transmitter device placed on the chest of the person. After that, the same simulation has been performed without the human body model. The parameters used for both simulations are the shown in Table 2.

**Figure 7.**
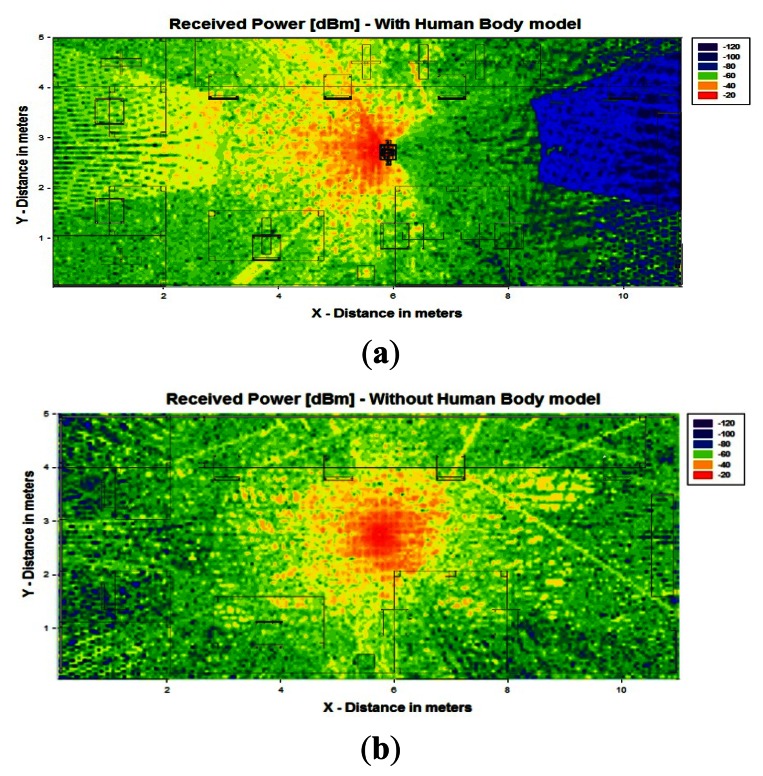
Spatial distribution of Received Power [dBm] for 1.30 meters height in the indoor scenario with (**a**) the presence of a human body model in the center (**b**) without the human body model.

Figure 7 shows the bidimensional distribution of received power for both cases, with and without the human body model in the indoor scenario with the transmitter location fixed at the point with coordinates (5.67 m, 4.67 m, 1.30 m), which correspond with the chest of the person.

**Figure 8.**
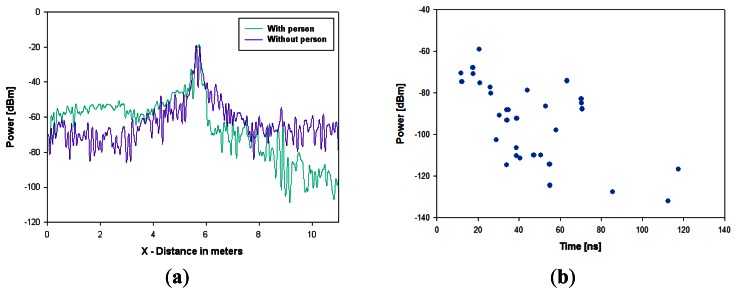
(**a**) Distribution of Power for Y = 4.67 meters along the X-axis for both cases, with and without the presence of a person (**b**) Power-Delay Profile at Point (3.41, 4.67, 1.35) meters in the indoor scenario.

The results obtained clearly show the strong influence in the signal degradation by the presence of the human body in it, as well as the topological and morphological dependence of the received power in relation to the indoor scenario itself. In order to further illustrate the dependence with the human body model and the spatial distribution, Figure 8(a) represents the received power distribution along the X-axis for Y = 4.67 meters, which correspond to the Y-position of the transmitter. It can be seen that with the presence of a person, the received power decreases in the rear location of the person. The strong dips in received power level are due to destructive addition of multipath components, described by statistically by fast fading.

To illustrate the relevance of multipath propagation, which is very significant in this type of scenarios, the power delay profile for a given point of the scenario has been computed and is shown in Figure 8(b). As it can be seen, there are a large number of echoes in the scenario, within a time span of approximately 15 ns to 120 ns, corresponding to trajectories for the rays from 4.5 meters to 36 meters including all the reflections. The large amount of echoes within such distance for the rays is coherent with the complexity of the scenario as well as the material properties at the frequency of operation of the Bluetooth link under analysis.

### 4.2. Measurement Results

**Figure 9.**
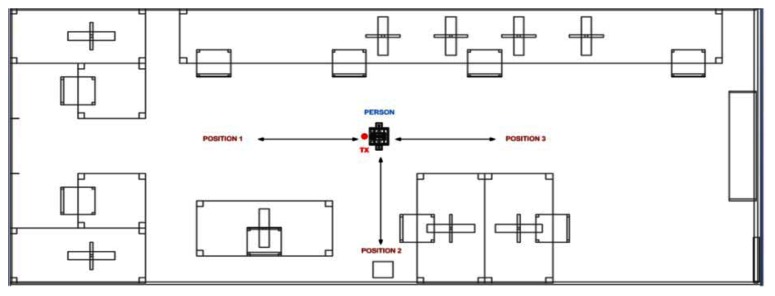
Layout of the measurement scenario.

To validate previous predictions, measurements in a real scenario with a real person have been performed. For this purpose, the experimental setup has been deployed in room N°2 of the Jerónimo de Ayanz Research Center of the Public University of Navarre, described in the previous section. The layout of the considered scenario is shown in Figure 9. All materials within the scenario have been taken into account for the simulation, like concrete for the walls and columns, glass for the windows, wood for the doors, considering their dielectric constant and conductivity for the given frequency of operation. Radiochannel measurements have been performed for three different positions, shown in Figure 9, with and without the presence of the human body model, with the transmitter fixed at the coordinate point (5.67 m, 4.67 m, 1.30 m).

**Figure 10.**
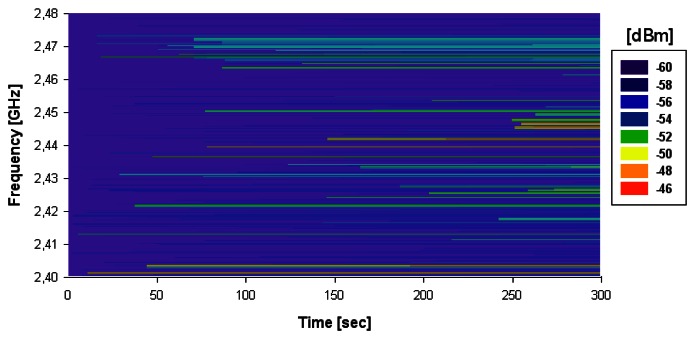
Measured spectrogram in 2.4 GHz ISM band, in the operating region of the Bluetooth wireless link of the HOLTIN-Smartphone connection.

The receiver antenna is from Antenova (Cambridge, United Kingdom), specifically a Picea 2.4 GHz Swivel Antenna, which is a vertical monopole with 0.82 dBi gain. The HOLTIN ECG recorder performs frequency hopping in each Bluetooth^®^ channel randomly to transmit the data. The storage capacity of the device is 32 Mbits, which is equivalent to two hours 75 minutes of continuous ECG signal, considering the sampling frequency of the HOLTIN which is 200 samples/s and 2 Bytes/sample. To visualize the frequency variation with time (and hence, the possible influence of external interference), a spectrogram has been measured in the scenario. Figure 10 shows the measured spectrogram in *Max Hold* mode, for Position 1 in the considered scenario, with the aid of portable spectrum analyzer (Agilent N9912 Field Fox, Agilent Technologies, Santa Clara, CA, USA).

**Figure 11.**
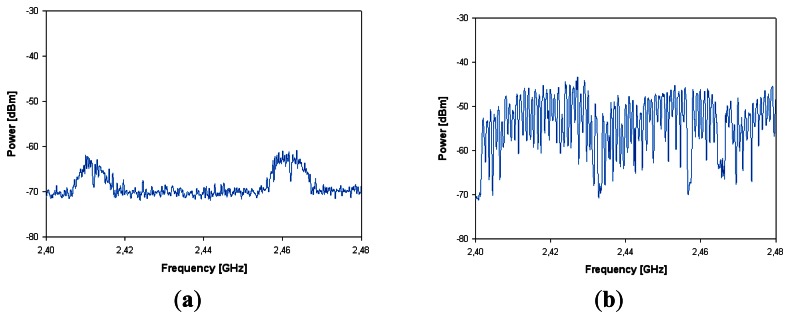
Measured spectrum without transmitting any data with the HOLTIN device (**b**) measured spectrum with the HOLTIN device is transmitting data.

In order to analyze pre-existent interference in the scenario, the spectrum without transmitting any data with the HOLTIN ECG recorder has been measured and is shown in Figure 11(a), where certain levels of interference in some channels of the 2.4 GHz ISM band are observed, which corresponds with a Wifi transmission in the room. Figure 11(b) shows the measured RF power in the same bandwidth with the HOLTIN ECG recorder transmitting data. Measurements have been performed during a continuous period of five minutes. From the measurement results, it can be seen that almost all channels transmit some data along the observed time span and therefore, some channels could be interfered with the pre-existent interfering signals previously shown.

**Figure 12.**
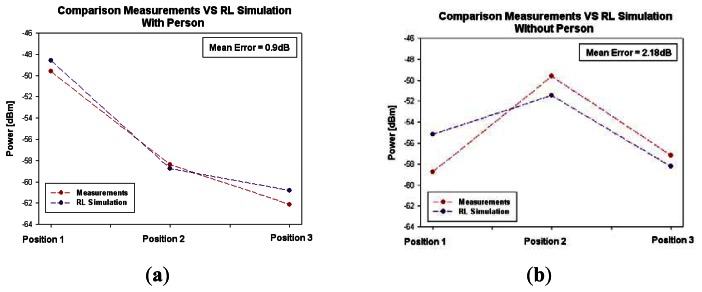
Comparison simulation versus measurements for different positions of the receiver in the considered scenario (**a**) With person (**b**) Without person.

Due to the presence of the pre-existent interference levels, the selected frequency of operation for comparison with simulations has been chosen to 2.44 GHz. As stated previously, two simulation calculations have been performed with the 3D ray launching algorithm for this frequency of operation. Figure 12 shows the comparison between simulation and measurements, for the three positions depicted in Figure 9. As it can be seen, they exhibit good agreement with a mean error around 2 dB for both cases. The differences are mainly due to geometrical approximations made in simulation, related with the inherent matrix calculation approach. It is also important to consider fast fading, which is a relevant effect in indoor environments due to multipath components which are very significant. It is observed that with the presence of the person, there are more variations in received power level between Position 1 and Position 3 of Figure 9. This is due to human body penetration losses which are present in the radio electric path and are dependent on human body position, which due to the material consideration embedded in the human body model implemented, is intrinsically considered.

**Figure 13.**
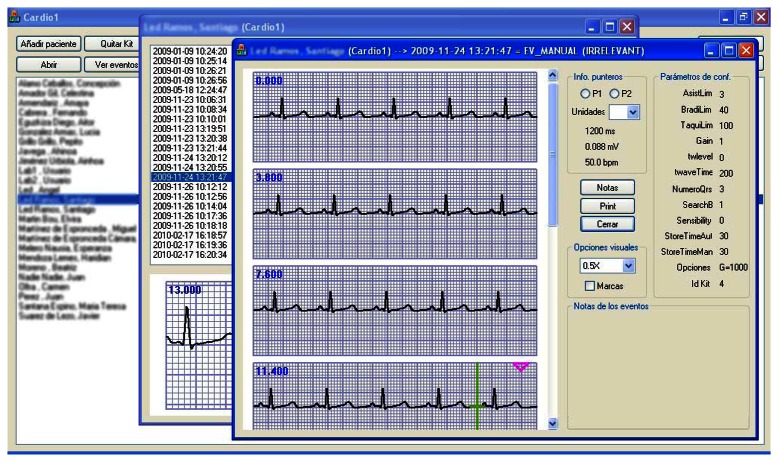
Real ECG waveform transmitted by the HOLTIN in the indoor scenario. Visualization in a proprietary software application for the HOLTIN platform has been developed specifically.

Once the assessment of the wireless channel between the HOLTIN ECG recorder and the gateway has been done in a typical indoor environment, it is shown that link quality is above nominal receiver sensitivity (−90 dBm). Accordingly, the relevant biomedical data is transported along the short range Bluetooth link (from HOLTIN device to the Smartphone acting as a gateway element) and later on via GPRS/UMTS Smartphone connection to the Application Server of the HOLTIN system. The image in Figure 13 is the actual representation that the medical specialist would remotely see, for example, in the hospital while the patient is located in his home.

## 5. Conclusions

In this paper, a novel platform for AECG monitoring implemented in-house, called HOLTIN, has been described and analyzed in terms of radiochannel quality. Indoor scenarios are one of the most common scenarios in which potential patients may use HOLTIN device for remote medical monitoring. Therefore, the analysis of the overall device's performance is needed. An in-house 3D Ray Launching code has been employed, in combination with an *ad-hoc* simplified human body model in order to estimate radiopropagation losses and hence sensitivity requirements of the short range Bluetooth link between the HOLTIN wearable ECG and a smartphone that acts as a gateway to the final monitoring application. Simulation results show the dependence with topology and morphology of the indoor scenario, in which material absorption as well as strong multipath components are mainly responsible for radio signal losses. Measurement results from the pre-existent signals in the indoor scenario, as well as from the operation of the short range communication link of the HOLTIN device have been performed, showing good agreement with the on-body measurements. Both the position on the human body and the relative transmitter-receiver location are relevant parameters in the overall performance of the device. The application of deterministic radioplanning techniques definitively aid in designing an optimal system layout, minimizing interference as well as reducing energy consumption. As an overall result, the HOLTIN system may represent a new step in U-Health services, reducing health assistance costs and increasing quality of life of patients.

## Figures and Tables

**Table 1.** Comparison of Features and Functionalities found in AECG systems.

	**HOLTIN**	**Holter Monitor**	**Insertable Loop Recorder** **(Reveal^®^Plus)**
Implantable	No	No	Yes
Ergonomics	+++	+	+++++
Automatic detection of cardiac events	Yes	No	Yes
Patient notification of syncopes	Yes	No	Yes
Service autonomy	Medical prescription Rechargeable battery 5 days (60 seconds cardiac events detected every 10 minutes)	24/48 hours	14 months
Storage type Storage capacity	Event recorder +++	Continuous ECG+	Event recorder +
Configurability	Yes	No	Yes
Availability of diagnostic data	Fast	Slow	Medium
Cost	++	+	+++++
Clinical utility	++++	++	+++++

**Table 2.** Parameters considered for the deterministic technique of ray launching.

Frequency	2.44 GHz
Transmitter power	0 dBm
Transmitter Antenna gain	1.89 dBi
Receiver Antenna gain	0.82 dBi
Horizontal plane angle resolution (ΔΦ)	1°
Vertical plane angle resolution (Δθ)	1°
Reflections	5
Cuboids resolution	3 cm × 3 cm × 3 cm
